# Iron and Neurodegeneration in Multiple Sclerosis

**DOI:** 10.1155/2011/606807

**Published:** 2011-02-10

**Authors:** Michael Khalil, Charlotte Teunissen, Christian Langkammer

**Affiliations:** ^1^Department of Neurology, Medical University of Graz, A-8036 Graz, Austria; ^2^NUBIN, Department of Clinical Chemistry, VU University Medical Center, 1007MB Amsterdam, The Netherlands

## Abstract

Increased iron deposition might be implicated in multiple sclerosis (MS). Recent development of MRI enabled to determine brain iron levels in a quantitative manner, which has put more interest on studying the role of iron in MS. Evidence for abnormal iron homeostasis in MS comes also from analyses of iron and iron-related proteins in CSF and blood and postmortem MS brain sections. However, it is not yet clear if iron accumulation is implicated in MS pathology or merely reflects an epiphenomenon. Further interest has been generated by the idea of chronic cerebrospinal venous insufficiency that might be associated with brain iron accumulation due to a reduction in venous outflow, but its existence and etiologic role in MS are currently controversially debated. In future studies, combined approaches applying quantitative MRI together with CSF and serum analyses of iron and iron-related proteins in a clinical followup setting might help to elucidate the implication of iron accumulation in MS.

## 1. Introduction

Iron is essential for normal neuronal metabolism, including mitochondrial energy generation and myelination [1, 2]. However, excessive levels of brain iron may exert iron-induced oxidative stress and thus lead to neurodegeneration [3]. During the process of normal aging, various regions of the brain, predominantly the basal ganglia, tend to accumulate nonhemin iron, which is primarily stored in the form of ferritin [4]. Increased iron deposition has been observed in various chronic neurological disorders, including multiple sclerosis (MS) [5].

Evidence for increased iron accumulation in MS is mainly derived from magnetic resonance imaging (MRI) and histopathologic studies; however, some information exists also from analyses of iron and iron-related proteins in cerebrospinal fluid (CSF) and blood. The following review summarizes current knowledge of increased brain iron accumulation in MS derived from (2) MRI, (3) histopathologic analyses, (4) studies on CSF and blood, and (5), finally, provides an outlook on potential therapeutic interventions. 

## 2. Magnetic Resonance Imaging

In several studies, evidence for increased iron accumulation, preferentially in deep gray matter areas of the brain, was mainly derived from the signal reduction on T2-weighted MR images [5]. 

First reports on a regionally signal reduction on T2-weighted brain MRI images in MS indicative of increased iron deposition were published by Drayer et al. [6] and Grimaud et al. [7]. 

Several studies then followed with a focus on the clinical implication of increased iron accumulation in MS. Increased deep gray matter T2 hypointensities were found to be correlated with disease duration [8, 9], physical disability [9–13], and cognitive impairment [14]. Clinical followup studies in MS revealed that baseline gray matter T2 hypointensities were associated with disability progression over time [12, 15]. Another consistent finding is that deep gray matter T2 hypointensity, suggestive of increased iron content, is correlated with brain atrophy [8, 16]. While this was evidenced in patients with definite MS, there is only little information available regarding the extent and clinical significance of increased iron deposition in patients with a clinically isolated syndrome. Ceccarelli et al. found only minor changes of signal reductions on T2-weighted images compared to healthy controls, and the extent did not predict conversion to clinically definite MS [17]. The approaches used in the studies mentioned above suffered from the methodological drawback of deducing iron concentrations from a visual grading of the reduction of signal intensity on T2-weighted images even though more recent studies have determined the extent of T2 hypointensity in a semiquantitative manner [8, 10, 14, 16]. 

In recent years, methodical development of MRI enabled to assess brain iron concentrations quantitatively. In addition, quantitative iron mapping by MRI offers a more sensitive discrimination of iron levels and, therefore, is especially advantageous in longitudinal studies and monitoring of long-term disease progression. 

**Figure 1:**
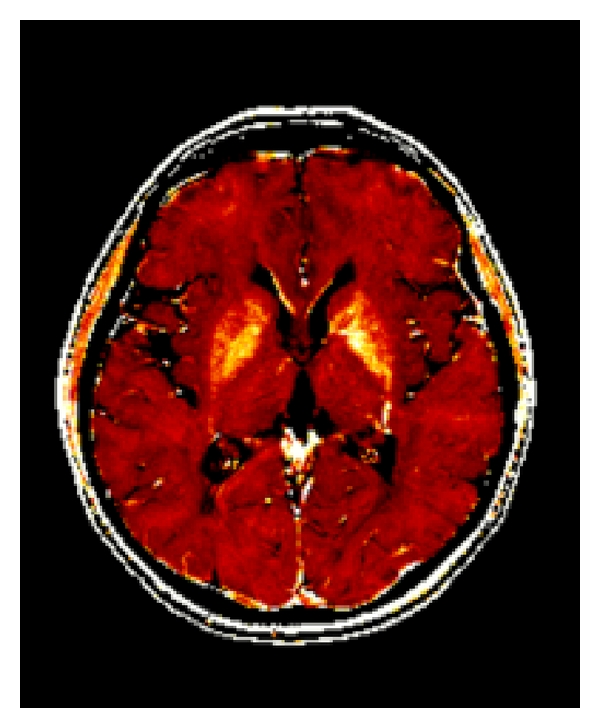
R2* map of a 50-year-old female MS patient. Higher iron concentrations in basal ganglia structures are reflected by brighter signal intensities.

The techniques utilized for quantitative iron mapping are mainly based on relaxation time mapping [18–20] (Figure 1) but also other approaches such as phase mapping [21, 22], magnetic field correlation [23], or direct saturation imaging [24] are applied. 

Susceptibility weighted imaging (SWI), a technique that takes advantage from the full complex MR signal by combining magnitude and phase images, has gained attention as a means to assess brain iron [25, 26]. However, the complexity of the postprocessing involved in SWI renders comparative studies challenging and remains an objective of research [27]. Quantitative susceptibility mapping (QSM) is an approach using solely phase images and produces susceptibility maps which are independent of the orientation of the tissue to the main magnetic field [28, 29]. Because paramagnetic iron is considered a main determinant of brain tissue susceptibility, QSM seems especially useful to assess brain iron. 

### 2.1. Validation of MRI Methods

Several methods have been proposed for the measurement of brain iron concentration; however, the majority of them lack validation and, therefore, the specificity and sensitivity of these techniques are not reliably known.

From theoretical considerations based on susceptibility models for brain tissue, it can be concluded that iron is a main determinant of susceptibility-induced contrast in MRI [30]. Several studies have indirectly investigated the relation of MRI parameters with iron by using the age-dependency of iron accumulation in the basal ganglia as reported in [4, 31]. 

Recently, high-pass filtered SWI phase images were compared to regional iron concentrations in postmortem tissue determined by synchrotron X-ray fluorescence and revealed a correlation between phase shifts and iron [32]. 

Other recent work acquired quantitative MRI directly after death from seven human brains and subsequently determined brain iron concentrations by using inductively coupled plasma mass spectrometry [33]. This study showed that the relaxation rates R2 and R2* can be used as sensitive and linear measures for brain iron concentration.

These quantitative MRI techniques together with a better understanding of pathophysiologic concepts of increased iron levels [1–3] have put more interest on elucidating the role of iron in MS. 

In recently performed studies on quantitative brain iron levels in MS, based on R2* relaxometry at 3 Tesla, increased iron levels have been found in patients with advancing MS compared to clinically isolated syndrome [20]. Using this validated quantitative technique, higher R2* levels in basal ganglia structures reflecting higher iron content were correlated with gray matter atrophy and also with T2-lesion volume [20]. These findings are supported by earlier studies where MRI T2 hypointensities suggestive of increased brain iron, preferentially located in deep gray matter areas, were linked to physical disability and gray mater atrophy in MS [8–10, 12, 34]. Further support comes from a followup study showing that MRI T2 shortenings in deep gray matter areas at baseline are predictive of the evolution of brain atrophy [16]. 

Apart from gray matter regions with known high iron levels (putamen, globus pallidus, caudate nucleus, substantia nigra, and red nucleus) efforts were made to investigate iron levels in white matter by MRI [22, 35, 36]. Using SWI, the phase values of MS lesions were investigated and compared to adjacent white matter [36]. However, compared with chemically determined iron concentrations of postmortem studies, the iron levels within MS lesions were not substantially altered than in reference white matter structures [4, 33]. Due to the confounding impacts of iron and myelin to MRI contrast generation, disease-induced alterations of iron levels in white matter need to be treated with caution and are an objective of ongoing research [37].

Further interest on iron deposition in MS has been generated by the idea of chronic cerebrospinal venous insufficiency (CCSVI) [38] that might be associated with the accumulation of iron in the brain due to a reduction in venous outflow [39, 40]. Following this hypothesis, CCSVI is postulated to be implicated in the etiology of MS. The underlying mechanism is believed to originate from increased iron accumulation in patients due to a reduced venous blood flow caused by constrictions of cerebral veins. This then leads to extravasation of erythrocytes with subsequent iron deposition [41], subsequently triggering inflammation-dependent tissue damage [42]. However, the existence of CCSVI as well as its etiologic role in MS are currently controversially debated [43], and there is an increasing amount of papers published now that challenge this hypothesis [44–47]. Furthermore, histopathologic studies do not provide clear evidence for extravasation of erythrocytes into lesions caused by increased intraluminal venous pressure [48–52]. 

## 3. Histopathology and Pathologic Significance of Increased Brain Iron

The normal anatomic and cellular age-dependent iron distribution within the brain, as described previously [4, 53, 54], should be considered when comparing with iron deposition in pathological conditions. 

Craelius et al. described positive iron staining in MS brain sections surrounding demyelinated plaques, myelinated white matter near the lesions, and within blood vessels of gray matter near the lesion [55]. Iron deposits were also described in the putamen and the thalamus [6], in macrophages and reactive microglia [56] and in normal-appearing white matter tissue [57]. Mehindate et al. showed that heme oxygenase 1, which is involved in regulating iron metabolism, was upregulated in astrocytes of MS spinal cord tissue [58].

The exact underlying mechanism by which brain iron accumulates in MS is not fully understood. Iron transport across the blood-brain barrier is dependent on iron transport proteins, predominantly by transferring receptors expressed on brain epithelial cells [59]. Other transporters may also facilitate iron transport across the blood-brain barrier, such as the divalent metal transporter (DMT) and the lactoferrin receptor [60]. 

**Figure 2:**
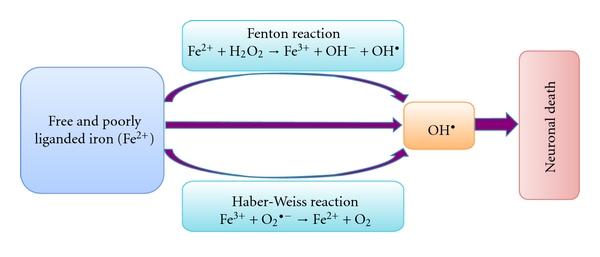
Generation of reactive and damaging hydroxyl radicals (OH^•^). Free Iron (Fe^2+^) reacts trough the Fenton reaction with hydrogen peroxide, leading to the generation of very reactive and damaging hydroxyl radicals (OH^•^). Superoxide can also react with ferric iron in the Haber-Weiss reaction leading to the production of Fe^2+^, which then again affects redox cycling. The highly reactive hydroxyl radicals lead to oxidative stress-induced lipid peroxidation, mitochondrial dysfunction, and increase in intracellular free-calcium concentration, and finally causing neuronal death.

It is also not yet clear if increased brain iron deposition is implicated in MS pathology or merely reflects an epiphenomenon [3, 61]. Potential toxic iron products may arise when hydrogen peroxide is formed by superoxide dismutase, which then reacts with free or poorly liganded iron (Fenton reaction [62]). Superoxide may also react with ferric iron through the Haber-Weiss reaction, producing Fe^2+^, which then again affects the redox cycling [1, 2] (Figure 2). 

The resulting highly reactive free hydroxyl radicals (OH^•^) interact with molecules leading to the production of other free radicals [63]. This leads to oxidative stress-induced lipid peroxidation, mitochondrial dysfunction, increase in intracellular free-calcium concentration, and finally causing cell dysfunction and death [62–64]. Because neuronal membrane lipids are rich in highly polyunsaturated fatty acid, they are susceptible to damage caused by lipid peroxidation [62, 63]. Iron itself can initiate and amplify lipid peroxidation [62, 63]. Several naturally produced antioxidants, such as alphatocopherol, may help to reduce oxidative stress-induced tissue damage [62]. 

## 4. Cerebrospinal Fluid and Blood

Only a limited number of studies have analyzed iron and iron-related protein levels in CSF and peripheral blood of MS patients. CSF ferritin levels were shown to be elevated in patients with chronic progressive active MS [65] and in patients with SPMS compared to controls [46, 57]. Another study showed that CSF ferritin levels were lower but within normal limits in patients with optic neuritis compared to patients with other neurologic diseases [66]. Similar levels of CSF ferritin were detected in RRMS patients compared to controls [57, 67]. In a recently performed cross-sectional and longitudinal study, CSF ferritin levels did not significantly change over a time period of 3 years, which also may argue against an etiologic role for CCSVI-related parenchymal iron deposition in MS [46].

Serum soluble transferring-receptor levels were significantly increased in MS compared to controls [68, 69], while serum ferritin levels were elevated in patients with chronic active MS only [68]. Conversely, analyses of iron status in two children with recurrent episodes of tumefactive cerebral demyelination revealed decreased serum iron and ferritin and constant iron supplementation was needed to prevent an iron deficiency state in both children [70]. 

## 5. Therapeutic Implications

On basis of pathophysiologic concepts implicating iron-induced tissue damage in MS, potential therapeutic interventions, including iron chelators, and inhibitors of iron-related oxidative stress and lipid peroxidation may have beneficial effects [3, 71, 72]. Several chelators are of putative therapeutic value in neurodegenerative disorders [73].

Studies on experimental autoimmune encephalomyelitis (EAE), the animal model of MS, showed that treatment with the iron chelator desferrioxamine reduced clinical and pathologic signs of EAE [74]. Deferiprone, an orally delivered iron chelator, ameliorated signs of EAE, an inhibited T-cell function [75]. However, a clinical trial testing the iron chelating drug desferrioxamine in chronic progressive MS patients failed to demonstrate any effects on disease progression [76]. A recent observation revealed that supplementing nonanaemic iron deficiency in two children with recurrent episodes of tumefactive demyelination leads to sustained remission [70].

In the future large randomized double-blinded multicenter studies are needed to elucidate the potential use of therapies targeting oxidative stress and lipid peroxidation in patients with MS. Quantitative MRI techniques and detailed monitoring of body-fluid iron and iron-related proteins levels should be included in such study protocols. 

## 6. Summary

In summary, increased iron deposition has been consistently reported to occur in MS, but its role in pathogenetic processes of this disease has not yet been completely clarified. Whether increased brain iron levels are also the cause or only the consequence of tissue destruction is still a matter of debate. Future longitudinal studies combining clinical disease status, quantitative MRI techniques sensitive for iron, and additional analyses of iron in CSF/serum and iron-related proteins (as well as iron regulator proteins), might help to unravel the implication of increased iron accumulation in MS. Quantitative MRI and histopathologic analyses of postmortem MS brains should complement these studies.
